# Submicron 3-D mass spectrometry imaging reveals an asymmetric molecular distribution on chemotaxing cells

**DOI:** 10.12688/f1000research.124273.1

**Published:** 2022-09-08

**Authors:** Anthony Castellanos, Richard H Gomer, Francisco Fernandez-Lima

**Affiliations:** 1 Chemistry and Biochemistry, Florida International University, Miami, Florida, 33199, USA; 2 Biology, Texas A&M University, College Station, Texas, 77843-3474, USA; 3 Biomolecular Science Institute, Florida International University, Miami, Florida, 33199, USA

**Keywords:** Dictyostelium, chemoattraction, mass spectrometry imaging, aggregation, molecular imaging

## Abstract

**Background:** *Dictyostelium discoideum* is a ~10 µm diameter unicellular eukaryote that lives on soil surfaces. When starved, *D. discoideum* cells aggregate into streams of cells in a process called chemotaxis. In this report, we studied *D. discoideum *cells during chemotaxis using 3D - mass spectrometry imaging (3D-MSI).

**Methods: **The 3D-MSI consisted of the sequential generation of 2D molecular maps using burst alignment coupled to delayed extraction time-of flight secondary ion mass spectrometry (TOF-SIMS) combined with a soft sputtering beam to access the different layers.

**Results: **Molecular maps with sub-cellular high spatial resolution (~300 nm) indicated the presence of ions at *m/z* = 221 and 236 at the front and sides, but reduced levels at the back, of cells moving toward of aggregation streams. The 3D-MSI also detected an ion at *m/z* = 240 at the edges and back, but reduced levels at the front, of aggregating cells. Other ions showed an even distribution across the cells.

**Conclusions: **Together, these results demonstrate the utility of sub-micron MSI to study eukaryotic chemotaxis.

## Introduction

Mass spectrometry techniques are immensely useful for the identification and characterization of molecular components in biological samples. ^1^^–^^3^ For instance, mass spectrometry imaging (MSI) permits the characterization of molecular components with high sensitivity and without the need for labels or pre-selection of molecules of interest. ^4^ Using MSI, molecules can be detected and localized simultaneously. ^5^ The spatial resolution of MSI is ultimately determined by the dimensions of the desorption probe (from tens of nanometers to hundreds of micrometers). ^6^^–^^8^ One such technique, Time of Flight Secondary Ion Mass Spectrometry (TOF-SIMS), particularly excels in the ability to provide good spatial resolution. Recent developments of surface probes for the analysis of biological samples have been based on the search for higher molecular desorption yields; for example, the introduction of cluster and nanoparticle probes for surface interrogation of biological surfaces with enhanced secondary ion yield and reduced damage cross section has permitted the detection of a broad range of chemical classes. ^9^^–^^15^ In addition, the combination of high spatial resolution cluster ion probes with reduced damage sputtering sources permits a 3D characterization of biological samples.

*D. discoideum* are ~10 μm diameter motile eukaryotic cells that during starvation aggregate into dendritic streams to form a fruiting body as a mechanism to disperse spores to start new colonies. During aggregation, relayed pulses of cyclic adenosine monophosphate (cAMP) activate signal transduction pathways that cause cells to move toward the source of cAMP. ^16^ The cells form dendritic aggregation streams, and because when the streams start forming, the single cells outside aggregation streams are consistently observed moving toward the streams, one can assign a front and back to a cell near a stream. Although there is some understanding of the distribution of selected cytoskeleton and signal transduction components in the moving cells, little is known about the general distribution of molecules in these cells.

In this report, we examine the distribution of chemical species associated with *D. discoideum* chemotaxis using a dual beam interrogation probe based on a 25 keV Bi _3_^+^ imaging probe combined with a 20keV Ar _1500_^+^ sputtering beam in spectral, imaging, and delayed-extraction imaging TOF-SIMS modes for high spatial resolution. We observe an asymmetric distribution of three prominent molecules or molecule fragments in the aggregating cells, illustrating the advantages of 3D-MSI.

## Methods

### Sample preparation

Aggregating *D. discoideum* Ax2 cells were prepared for mass spectrometry surface analysis as described previously. ^17^ Briefly, mid-log cells (1-2 × 10 ^6^ cells/ml) grown in shaking culture at 21°C in Formedium HL-5 were collected by centrifugation at 1,500 × g for 4 minutes, followed by three rounds of resuspension in deionized water, and collection by centrifugation at 1,500 × g for 4 minutes as described previously. ^18^ Following the final collection step, cells were resuspended in deionized water to a final concentration of 5 × 10 ^6^ cells/ml. To initiate chemotaxis on a surface, 80 μl droplets of the cells were spotted onto 1×1 cm gold-coated silicon chips (Sigma Aldrich). After allowing cells to settle for 30 minutes, 40 μl of the overlaying deionized water was removed and the Au/Si chips were placed in a humid box at 21°C for 17 hours. After verifying the existence of aggregation streams, Au/Si chips were gently drained, freeze-dried and stored over a CaCl _2_ desiccant at room temperature prior to mass spectrometry analysis. This procedure was repeated for all the replicates (n = 3), and high reproducibility of stream formation, surface coverage, and cell distribution was observed.

### Mass spectrometry

Mass spectrometry experiments were performed utilizing a TOF SIMS ^5^ instrument (ION-TOF, Münster, Germany) retrofitted with a liquid metal ion gun analytical beam for high spatial resolution (25 keV Bi _3_^+^), an argon cluster sputtering gun (20 keV Ar _1500_^+^), and an electron flood gun to reduce surface charging during mass spectrometry analysis. The TOF-SIMS instrument was operated in spectral (“high current bunched”, HCBU), imaging (“burst alignment”, BA) and delayed-extraction imaging (BA-DE) modes as described previously. ^19^^–^^21^ The tradeoff between the three modes is the mass resolving power, spatial resolution and secondary ion collection efficiency. ^22^^,^^23^ Two dimensional maps (2D-MSI) were collected by rastering the primary 25 keV Bi _3_^+^ beam over the field of view of interest. In spectral HCBU mode, mass spectra were collected in positive and negative mode with a typical spatial resolution of 1.2 μm, a mass resolving power of m/Δm= ~5,000 at m/z = 591 (Au _3_^+^) and total ion dose ~1-4x10 ^12^ ion/cm ^2^. Tridimensional maps were collected by sequentially collecting two dimensional maps using the BA imaging (or BA-DE) modes of the 25 keV Bi _3_^+^ imaging probe followed by a sputtering cycle using the 20 keV Ar _1500_^+^ sputtering beam. The BA and the BA-DE imaging modes provided high spatial resolution (~300 nm) and nominal (m/Δm= ~200) and high (m/Δm = ~4,000) mass resolutions at m/z= 197, respectively. The main differences between BA-DE and BA were the higher mass resolution and longer acquisition times (~4×) for BA-DE relative to BA. The typical total ion doses were 3×10 ^12^ ions/cm ^2^ for the 25 keV Bi _3_^+^ imaging probe and 5×10 ^12^ ions/cm ^2^ for the 20 keV Ar _1500_^+^ sputtering beam during BA and BA-DE analysis. The tridimensional experiments comprised 300-500 two dimensional scans. Replicate measurements (n = 3) were performed on independent chips.

### Data analysis

TOF-SIMS data processing was performed using SurfaceLab 6 software (ION-TOF, Münster, Germany). Two dimensional maps were processed from the BA and the BA-DE imaging modes at the level of unit and 0.1 *m/z*, respectively. Except when noted, two dimensional maps were not binned. Peak signals were exported in the.BIF3D format, binned 5X and imported into ZCORRECTORGUI. The Total Counts Threshold Value was set to 75 before initializing data.

## Results

**Figure 1.**
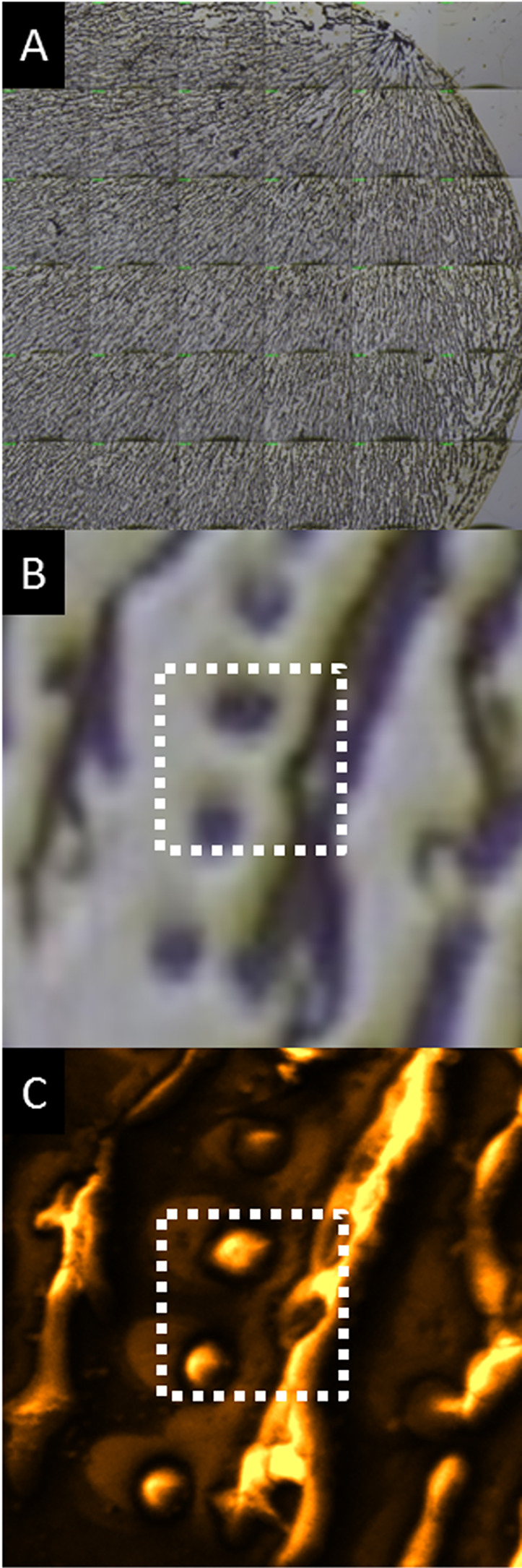
Optical (A, 4500×4500 μm and B, 120×120 μm field of view) and secondary ion total images (C, 120×120 μm field of view) of aggregating *D. discoideum* cells on a Au/Si substrate. Data are representative of 3 independent experiments.

**Figure 2.**
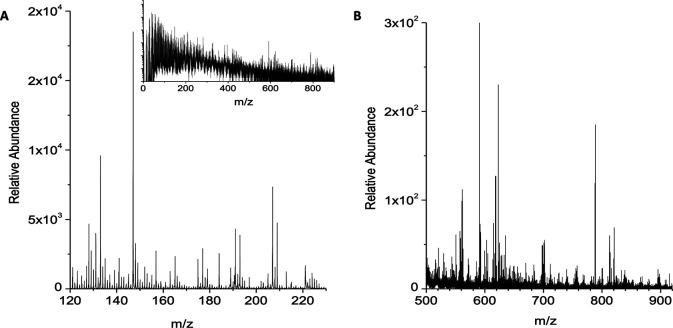
Representative TOF-SIMS positive ion HCBU mode spectrum of *D. discoideum* cells developed on a Au/Si substrate (250×250 μm field of view). A and B show selected regions of the spectrum; insert shows the entire spectrum. Data are representative of 3 independent experiments.

Visual inspection of *D. discoideum* cells developed on the Au/Si chips showed that cells were aggregating ( Figure 1A and B). Closer inspection showed a recurrent trend of single cells moving towards a recently formed stream. Cell integrity and morphology was preserved during the drying process, burst cells were not observed, and sample reproducibility was very high. A good correlation was observed between the optical and the TOF-SIMS images using HCBU, BA and BA-DE modes (see for example Figure 1B and C). Summed spectra of the TOF-SIMS images in HCBU mode were used to detect characteristic molecular ion signals from the single cells and from the streams in the process of aggregation. The *m/z* = 0-230 region of the spectra showed ions at *m/z* of 139.00, 160.98, 166.03, 184.07, 224.10 detected on the cells, and the characteristic substrate signal of Au ^+^ ion at 196.97 detected in regions where no cells were present ( Figure 2A). The *m/z* = 500-950 region showed ions at *m/z* of 518.30, 520.32, 740.50, 742.51, and 908.58 detected on the cells, as well as characteristic Au/Si chip substrate gold cluster ions at *m/z* 590.90 (Au _3_^+^), 787.87 (Au _4_^+^), and 418.94 (Au _2_C _2_H ^+^) detected in regions where no cells were present ( Figure 2B). These observations are in good agreement with our previous FT-ICR SIMS analysis of *D. discoideum* cells developed on Au/Si chips. ^17^ There were no obvious differences in the HCBU mode *m/z* signals from single cells and from streams, and the ~1.2 μm resolution *m/z* signals showed an apparently homogenous distribution on the ~10 μm diameter cells and the streams of cells.

**Figure 3.**
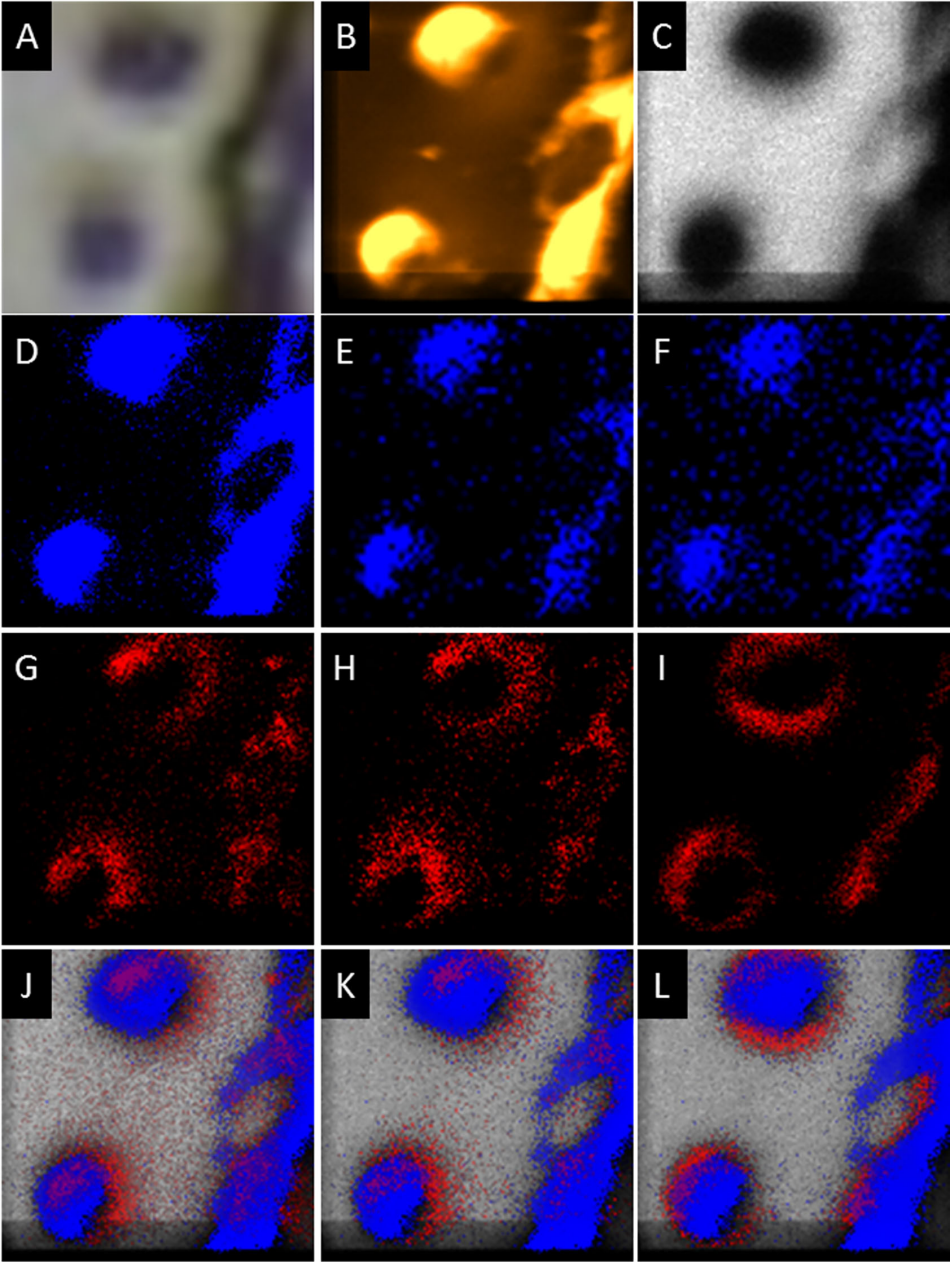
A) Optical image of aggregating *D. discoideum* cells (40×40 μm field of view). B) TOF-SIMS BA mode total secondary ion image of the same field. C-I) TOF-SIMS BA mode images pf this field at *m/z* C) 591 (Au _3_^+^ substrate, light grey), D) 184, (Blue), E) 518 (Blue), F) 740 (Blue), G) 221 (Red), H) 236 (Red), and I) 240 (Red). J-L) Overlays of 591 (Au _3_^+^ substrate, light grey), 184 (Blue), and J) 221 (Red), K) 236 (Red), and L) 240 (Red). Data are representative of 3 independent experiments.

**Figure 4.**
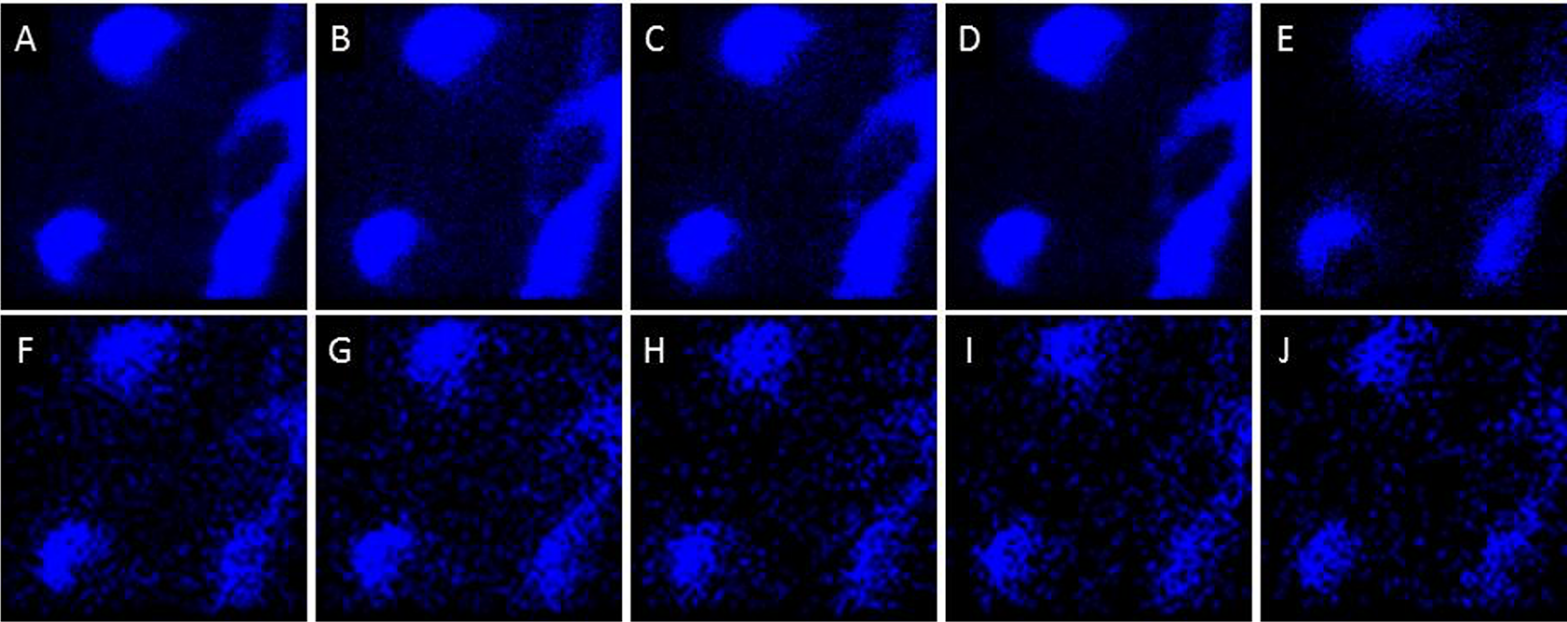
Distribution of additional ions in the 40×40 μm field of view in Figure 3 from the tridimensional analysis using BA mode at *m/z* A) 125, B) 147, C) 166, D) 184, E) 224, F) 518, G) 520, H) 740, I) 742, and J) 908. Images F-J are binned x4 for clarity. Data are representative of 3 independent experiments.

Higher spatial resolution imaging (~300 nm resolution) was performed over smaller fields of view (40×40 μm; dashed squares in Figure 1B and C) using BA and BA-DE modes to study the distribution of molecular components in the cells and in the streams. The analysis has the added benefit that multiple 2-dimensional scans can be added, thus increasing the signal to noise and increasing the probability of observing lower abundance components. ^24^ There appeared to be asymmetric distributions on cells and streams of ions at *m/z* = 221, 236 and 240, while all the other signals were assigned to either Au/Si chip substrate peaks or components evenly distributed across the cells and the streams ( Figures 3, 4, and S1). The signals at *m/z* = 221 and 236 were concentrated at the leading edge and sides of cells moving toward streams and at the borders/edges of streams, while the signal at *m/z* = 240 was concentrated at the edges of cells and the sides of streams ( Figures 3, 4, and S1).

## Discussion

In this report, we observed asymmetric distributions of molecular components at *m/z* = 221, 236 and 240 on chemotaxing cells using 3D-MSI. We also observed several other molecular components that showed an even distribution on cells. Beam effects related to the incident angle of the primary beam during TOF-SIMS analysis (e.g., shadow effects) were not the cause of the asymmetric distributions, since they are expected for all the observed secondary ion signals and not a subset of them.

A challenge during 3D-MSI is the high diversity of biological molecules responsible for the ion signals, and complementary mass spectrometry tools would be needed for candidate assignments. One possibility is to perform tandem mass spectrometry for structural identification; however, due to the low secondary ion yields observed at the *m/z* of interest using TOF-SIMS, this approach is almost unpractical at the spatial resolution level required despite recent advances in MS/MS SIMS instrumentation. ^14^^,^^25^ Alternatively, ultrahigh resolution mass spectrometry might permit assignments based on accurate mass measurements. ^17^^,^^26^

## Ethical approval

*Dictyostelium discoideum* is a unicellular invertebrate eukaryotic microbe, so no human subjects or vertebrate animal ethical approval was needed for these studies. *Dictyostelium discoideum* is a BSL-1 organism, and all work with live cells was done under a Texas A&M Institutional Biosafety Committee-approved BSL-1 protocol (IBC2018-155, with the most recent re-approval on March 22, 2022).

## Data availability

### Underlying data

Figshare: 20150124 DictyPT2 S4 Bi3+ SM+FGON 0.21pA 3scans FOV 200 007.itm, https://doi.org/10.6084/m9.figshare.20346711.v1. ^27^

Figshare: 20150124 DictyPT2 S4 Bi3+ SM+FGON 0.21pA 3scans FOV 200 007_0.ita, https://doi.org/10.6084/m9.figshare.20346705.v1. ^28^

Figshare: 20151023 ND7 5 3D FI-DE+ 0.04pA Ar1500 0.48nA FG ON 015_0.ita, https://doi.org/10.6084/m9.figshare.20346696.v1. ^29^

Figshare: 20140814 Dicty Stream #2 ROI E6 Bi3 Primary + mode 019 0.11pA PI.itm, https://doi.org/10.6084/m9.figshare.20346690.v1. ^30^

Figshare: 20140814 Dicty Stream #2 ROI E6 Bi3 Primary + mode 020 0.11pA PII_0.ita, https://doi.org/10.6084/m9.figshare.20346726.v1. ^31^

Figshare: 20140814 Dicty Stream #2 ROI E6 Bi3 Primary + mode 020 0.11pA PII.itm, https://doi.org/10.6084/m9.figshare.20346723.v1. ^32^

Figshare: 20140814 Dicty Stream #2 ROI E6 Bi3 Primary + mode 019 0.11pA PI_0.ita, https://doi.org/10.6084/m9.figshare.20346717.v1. ^33^

Figshare: 20151023 ND7 5 3D FI-DE+ 0.04pA Ar1500 0.48nA FG ON 015.itm, https://doi.org/10.6084/m9.figshare.20346741.v1. ^34^

### Extended data

Figshare: Figure 2.tif, https://doi.org/10.6084/m9.figshare.20347146.v1. ^35^

Figshare: Figure 3.tif, https://doi.org/10.6084/m9.figshare.20347152.v1. ^36^

Figshare: Figure 1.tif, https://doi.org/10.6084/m9.figshare.20347149.v1. ^37^

Data are available under the terms of the Creative Commons Attribution 4.0 International license (CC-BY 4.0).
