# Supplementary material for: Submicron 3-D mass spectrometry imaging reveals an asymmetric molecular distribution on chemotaxing cells
Source: F1000Res. 2022 Sep 8;11:1017. [Version 1] doi: 10.12688/f1000research.124273.1 (PMC9947426; doi:10.12688/f1000research.124273.1)
Supplement: Peer review history

# Peer review: Reviewer response for version 1

**DOI:** 10.5256/f1000research.136459.r157161

**Published:** 2023-02-22

**Authors:** Jonathan Chubb

**Affiliations:** 1 Laboratory for Molecular Cell Biology and Department of Cell and Developmental Biology, University College London, London, UK

This study uses spatial mass spectrometry approaches to explore molecular heterogeneity across cells during the aggregation phase of Dictyostelium. The study identified specific signals which showed striking variability along the cell axis, with orientation towards a likely source of chemoattractant. This is really impressive and the approach might be very useful, as it is unbiased, rather than based on prior knowledge of the signalling pathways identified using more traditional molecular approaches. The study is mostly very clearly described, even for a non-expert in mass spectrometry, and well presented.

 I have a few comments that the authors may wish to consider:

 It took a while to work out what the high magnification images in Figure 1 represent. It would be good to expand the narrative in the legend and /or label the figure to orient the reader. It also took a while to realise I was looking at streaming- again- some expanded legend material or some marking on the figure to show the aggregation centre/direction of migration would help.

 In Figure 3, perhaps it would help the reader to know how you assigned the polarity of the cells from the optical image (they are somewhat round) although the ion images themselves are compelling. In addition- this is a 3D approach- would it be possible to have a 3D representation of the ion images in the main text eg. Fig 3G-I? This might be informative, even in the absence of molecular identity. I do not know the method, so maybe a "z-stack" cannot be done.

 In the discussion, I would have liked to see a little thinking about what types of molecules might generate these distributions. This would align the study more to the literature, and so bring it more attention. For example are the sharp gradients- eg. Fig3 G-I, compatible with what is known about the distribution of actin, cAMP, or something else?

Is the work clearly and accurately presented and does it cite the current literature?

Yes

If applicable, is the statistical analysis and its interpretation appropriate?

Yes

Are all the source data underlying the results available to ensure full reproducibility?

Yes

Is the study design appropriate and is the work technically sound?

Yes

Are the conclusions drawn adequately supported by the results?

Yes

Are sufficient details of methods and analysis provided to allow replication by others?

Yes

Reviewer Expertise:

Dictyostelium, signalling, gene expression.

I confirm that I have read this submission and believe that I have an appropriate level of expertise to confirm that it is of an acceptable scientific standard.

# Peer review: Reviewer response for version 1

**DOI:** 10.5256/f1000research.136459.r149946

**Published:** 2022-10-04

**Authors:** Serge Della Negra

**Affiliations:** 1 CNRS-IN2P3, Institut de Physique Nucléaire, Orsay, France

This manuscript presents a study by mass spectrometry imaging of *Dictyostelium discoideum* aggregation. *Dictyostelium discoideum* is a model organism used to investigate many cellular processes including chemotaxis, cell motility, cell differentiation, and human disease pathogenesis. Key questions can be addressed using *Dictyostelium* as a model organism. The goal of the authors is the study of  D. discoideum cells during chemotaxis using 3D - mass spectrometry imaging (3D-MSI). The instrument used for this research is IONTOFV LMIS Bismuth Source and Argon Cluster Gun.

 The presentation of the goal is clear and also the different experimental steps with the preparation of the samples already studied by this team, as well as the imaging techniques that are allowed by the IONTOFV instrument. It is clear that the authors know their instrumentation well with the associated bibliography. Their results and the ionic images thus obtained benefit from the best techniques with the BA-DE and the improvement of the signal-to-noise ratio permitted by the use of 3D MSI approach. The highlighting of two different regions with a peripheral concentration probably membranous is clearly imaged.

 Personally, I would like the authors to present the mass area of interest (MW: 220-241 daltons) in high resolution and also the precise masses and uncertainties.

 The authors are not optimistic in their conclusions for future determinations of these molecules, despite the new M6 and hybrid developments of IONTOF. I would have liked to have some values knowing that an accumulation of data on several ROI is always possible.

 Regarding the chemotaxis objective, the authors could specify some assumptions, these molecules are in relation with:

 -the recognition process;

 -the polarisation process;

 -or the displacement process.

 Dictyostelium has cAMP receptors uniformly distributed on the surface of its plasma membrane. The receptors are coupled to heterotrimeric G proteins, the activation of different signalling pathways are described ^1^. Is there a link with these processes? It should be noted that Dictyostelium cells use cAMP as described in this paper which allows subsequent cells to reach the centre of the aggregate. There is an asymmetry between where the concentration of chemoattractant is greatest, i.e. at the front of the cell, and where the concentration of chemoattractant is lowest. There are illustrations of this polarisation of chemotactic cells ^2^. This requires knowledge of the direction of progression. Numerous studies on proteins and the different signalling steps have been described and indications can be found.

 For mobility there are also studies that have demonstrated a three-phase cycle:

 1) The emission of protrusions at the front of the cell resulting in membrane deformation. These forces are obtained thanks to the polymerisation of actin filaments.

 2) The adhesion of these protrusions to the substrate. The adhesion of the protrusions to the substrate to pull the cell forward. Cellular adhesion involves many molecular assemblies, including integrin-like proteins.

 3) Detachment and retraction of the cell's rear end. Once the cell is well stretched on the surface, it must contract before it can extend again. In particular, it involves assemblies of myosin II and actin filaments. Same question as above?

 I don't know if these relationships are possible but if the observation described in this article is related to aggregation it would be necessary to show the image of isolated cells (this point will demonstrate the link with the aggregation of these molecules in the membrane) and cells in the process of aggregation or to include in the conclusion to continue these studies with these points.

 Regardless of my questions and suggestions this article can be published with a minimum of modification. Especially if possible a conclusion or discussion in relation to the knowledge obtained elsewhere about this chemotaxis would be interesting.

Is the work clearly and accurately presented and does it cite the current literature?

Yes

If applicable, is the statistical analysis and its interpretation appropriate?

Yes

Are all the source data underlying the results available to ensure full reproducibility?

Yes

Is the study design appropriate and is the work technically sound?

Yes

Are the conclusions drawn adequately supported by the results?

Partly

Are sufficient details of methods and analysis provided to allow replication by others?

Yes

Reviewer Expertise:

My main activity for many years concerns the fundamental research on particle-solid interaction. These fundamental studies, which covered a large energy range from keV to GeV for atomic ions, led me to develop new molecular and metallic cluster ion beams. I obtained the  first high energy fullerenes beams which I accelerated to 40 MeV and metal aggregates using LMIS sources in the keV to 10 MeV range in the early 1990's. Again, fundamental studies on secondary emission and material modification induced by these complex beams led me to a new source development for accelerating metallic nanoparticles in the 2000s.  At Orsay I led a new project Andromede: High velocity molecule and Nano-particle projectiles : Probe of nano domains and environmental nano objects. This project is focused on the following scientific  area: Nano-particles, Surface analysis, Mass spectrometry, Biology, Astrochemistry, catalysis, solid-particle Interaction.

I confirm that I have read this submission and believe that I have an appropriate level of expertise to confirm that it is of an acceptable scientific standard.

## References

[1] : The signal to move: D. discoideum go orienteering. *Science* .2003;300(5625) : 10.1126/science.1085439 1525-7 10.1126/science.1085439 12791977

[2] : PI 3-Kinases and PTEN. *Cell* .2002;109(5) : 10.1016/S0092-8674(02)00765-1 541-544 10.1016/S0092-8674(02)00765-1 12062096
